# Supplementary material for: Mercury methylating microbial communities of boreal forest soils
Source: Sci Rep. 2019 Jan 24;9:518. doi: 10.1038/s41598-018-37383-z (PMC6345997; doi:10.1038/s41598-018-37383-z)
Supplement: Supplementary file 1 — Supporting Information [file 41598_2018_37383_MOESM1_ESM.docx]

Mercury methylating microbial communities of boreal forest soils

Jingying Xu^1, *^, Moritz Buck^1^, Karin Eklöf ^2^, Omneya O. Ahmed^1^, Jeffra K. Schaefer^3^*,* Kevin Bishop^2^, Ulf Skyllberg^4^, Erik Björn^5^, *S*tefan Bertilsson^1^, Andrea G. Bravo^1, 6, *^

*^1^ Department of Ecology and Genetics, Limnology, Uppsala University, Uppsala, Sweden*

*^2^ Department of Aquatic Sciences and Assessment, Swedish University of Agricultural Sciences, Uppsala, Sweden*

*^3^ Department of Environmental Sciences, Rutgers University, New Brunswick, New Jersey, USA*

*^4^ Department of Forest Ecology and Management, Swedish University of Agricultural Science, Umeå, Sweden*

*^5^ Department of Chemistry, Umeå University, Umeå, Sweden*

^6^ *Department of Marine Biology and Oceanography, Institut de Ciències del Mar, Consejo Superior de Investigaciones Científicas, Barcelona, Catalunya, Spain*

*corresponding authors: jingying.xu@ebc.uu.se; jandriugarcia@gmail.com

**Figure S1.** Overall coverage of the rarefied forest bacterial community (left) and Hg^(II)^ methylating microbial community (right) reflected in the combined richness detected for random subsets of analysed samples.

**Figure S2**. Maximum likelihood phylogenetic tree representing phylogenetic distribution of *hgcA* based most abundant OTUs from soil samples at Swedish sites. Inside the boxes are presented the number of OTUs. Outside the boxes the names of some reference sequences from GenBank.

**Table S1.** Site characteristics

| **Catchment** | **Region** | **Treatment** | **Years since harvest** | **Above or below highest postglacial coastline** | **Approximate O horizon thickness (cm)** |
| --- | --- | --- | --- | --- | --- |
| SP1 | Örebro | Stem harvest | 6 years | Above | >20 |
| SH1 | Örebro | Stem & Stump harvest | 6 years | Above | >20 |
| R1 | Örebro | Mature forest | >30 years | Above | >20 |
| SP2 | Balsjö | Stem harvest | 6 years | Above | <10 |
| SP3 | Balsjö | Stem harvest | 6 years | Below | <10 |
| R2 | Balsjö | Mature forest | >30 years | Above | <10 |
| SH2 | Strömsjöliden | Stem & Stump harvest | 2 years | Above | <5 |
| R3 | Strömsjöliden | Mature forest | >30 years | Above | <5 |

**Table S2.** Soil geochemistry in three studied regions (THg: total Hg; MeHg: methylmercury, n_Örebro_ = 75, n_Balsjö_ = 75, n_Strömsjöliden_ = 50; ±SD)

|  | Örebro  (59°10´16.39˝N 14°34´3.01˝E) | | Balsjö  (64° 1´37˝ N 18° 55´43˝E) | Strömsjöliden  (64°6´48˝N 19°7´36˝E) |
| --- | --- | --- | --- | --- |
| Water Content (%) | | 31.6 ± 10.9 | 34.8 ± 9.3 | 31.6 ± 8.7 |
| THg (ng/g) | 247.5 ± 132.9 | | 178.8 ± 77.1 | 244.5 ± 138.3 |
| MeHg (ng/g) | 1.4 ± 1.6 | | 0.8 ± 0.9 | 2.1 ± 1.9 |
| % MeHg | 0.7 ± 1.0 | | 0.6 ± 1.5 | 1.2 ± 1.0 |
| N (%) | 1.3 ± 0.7 | | 1.5 ± 0.6 | 1.1 ± 0.8 |
| C (%) | 32.4 ± 14.7 | | 43.9 ± 14.8 | 21.4 ± 14.7 |
| S (%) | 0.2 ± 0.1 | | 0.3 ± 0.1 | 0.2 ± 0.1 |
| C/N | 28.0 ± 9.9 | | 30.0 ± 5.8 | 20.6 ± 5.0 |
| C/S | 163.4 ± 69.0 | | 179.2 ± 69.4 | 128.8 ± 53.7 |

**Table S3.** Pearson correlation coefficients between geochemical parameters across all three regions (n_Örebro_ = 75, n_Balsjö_ = 75, n_Strömsjöliden_ = 50, *p* < 0.001). Significant (R ≥ 0.7) and moderate (0.5≤R<0.7) correlations appear in bold.

|  | Water (%) | THg (ng/g) | MeHg (ng/g) | %MeHg | N (%) | C (%) | S(%) | C/N |
| --- | --- | --- | --- | --- | --- | --- | --- | --- |
| Water (%) | 1. |  |  |  |  |  |  |  |
| THg (ng/g) | 0.236 | 1. |  |  |  |  |  |  |
| MeHg (ng/g) | 0.235 | 0.116 | 1. |  |  |  |  |  |
| % MeHg | 0.031 | -0.247 | **0.639** | 1. |  |  |  |  |
| N (%) | **0.688** | **0.522** | 0.081 | -0.229 | 1. |  |  |  |
| C (%) | **0.579** | 0.367 | -0.151 | -0.339 | **0.854** | 1. |  |  |
| S (%) | **0.550** | 0.221 | 0.124 | -0.100 | **0.741** | **0.661** | 1. |  |
| C/N | -0.277 | -0.297 | -0.400 | -0.114 | -0.333 | 0.115 | -0.174 | 1. |
| C/S | 0.022 | 0.138 | -0.287 | -0.282 | 0.056 | 0.302 | -0.331 | 0.429 |

**Table S4.** Soil geochemistry in 34 hotspots (THg: total Hg; MeHg: methylmercury; n_Örebro_ = 11. n_Balsjö_ = 4. n_Strömsjöliden_ = 19; ±SD)

| Regions | Örebro | Balsjö | Strömsjöliden |
| --- | --- | --- | --- |
| Water Content (%) | 38.4 ± 13.3 | 29.6 ± 14.4 | 23.6 ± 8.7 |
| THg (ng/g) | 189.6 ± 103.9 | 77.5 ± 99.2 | 107.9 ± 76.5 |
| MeHg (ng/g) | 3.9 ± 2.1 | 2.4 ± 2.4 | 2.3 ± 1.8 |
| %MeHg | 2.4 ± 1.3 | 4.5 ± 5.1 | 2.1 ± 0.7 |
| N weight (%) | 1.6 ± 1.0 | 0.7 ± 1.2 | 0.3 ± 0.3 |
| C weight (%) | 32.3 ± 20.0 | 15.5 ± 21.5 | 7.3 ± 5.7 |
| S weight (%) | 0.4 ± 0.2 | 0.2 ± 0.2 | 0.1 ± 0.1 |
| C/N | 22.2 ± 5.9 | 40.9 ± 22.8 | 22.5 ± 6.6 |
| C/S | 93.1 ± 33.9 | 96.3 ± 1.5 | 116.4 ± 52.5 |

**Table S5.** Pearson correlations between Hg methylating families, S% and C/S in hotspots (n = 34) based on *hgcA* sequencing. Moderate (0.5≤R<0.7) to weak (0.3≤R<0.5) correlations were in bold, significant level *p* < 0.001.

|  | *Desulfarculaceae* | *Desulfovibrionaceae* | *Desulfuromonadaceae* | *Methanomassiliicoccaceae* | *Methanoregulaceae* | *Ruminococcaceae* |
| --- | --- | --- | --- | --- | --- | --- |
| *S %* | **0.54** | **0.59** | **0.54** | **0.51** | **0.59** | 0.11 |
| *C/S* | -0.19 | -0.28 | **-0.39** | **-0.30** | -0.09 | **-0.39** |
| *Desulfarculaceae* |  | 0.10 | **0.83** | 0.14 | **0.31** | 0.06 |
| *Desulfovibrionaceae* |  |  | 0.29 | **0.82** | **0.70** | 0.16 |
| *Desulfuromonadaceae* |  |  |  | 0.25 | 0.11 | **0.39** |
| *Methanomassiliicoccaceae* |  |  |  |  | **0.62** | -0.03 |
| *Methanoregulaceae* |  |  |  |  |  | -0.05 |

**Table S6.** Pearson correlations between geochemical parameters in hotspots (n = 34. *p* < 0.001) Significant (R ≥ 0.7) and moderate (0.5≤R<0.7) correlations appear in bold.

|  | Water (%) | THg (ng/g) | MeHg (ng/g) | %MeHg | N (%) | C (%) | S(%) | C/N |
| --- | --- | --- | --- | --- | --- | --- | --- | --- |
| Water (%) | 1. |  |  |  |  |  |  |  |
| THg (ng/g) | **0.7** | 1. |  |  |  |  |  |  |
| MeHg (ng/g) | **0.563** | **0.627** | 1. |  |  |  |  |  |
| %MeHg | -0.095 | -0.497 | 0.125 | 1. |  |  |  |  |
| N (%) | **0.871** | **0.771** | **0.599** | -0.27 | 1. |  |  |  |
| C (%) | **0.845** | **0.722** | **0.55** | -0.243 | **0.988** | 1. |  |  |
| S (%) | **0.723** | **0.651** | 0.345 | -0.25 | **0.854** | **0.86** | 1. |  |
| C/N | -0.342 | **-0.583** | -0.386 | **0.532** | -0.377 | -0.274 | -0.272 | 1. |
| C/S | 0.005 | 0.113 | 0.047 | -0.15 | 0.025 | 0.059 | -0.301 | 0.077 |

**Table S7.** Barcoded primers used for *hgcA* gene pool

| **Sample** | **Code in pool** | **Forward index sequence** | **Reverse index** |
| --- | --- | --- | --- |
| 19 | HS1 | ACTGCATA | TCTAGGCA |
| 36 | HS2 | AAGGAGTA | TCGCCTTA |
| 37 | HS3 | AAGGAGTA | CTAGTACG |
| 38 | HS4 | AAGGAGTA | TTCTGCCT |
| 45 | HS5 | AAGGAGTA | GCTCAGGA |
| 50 | HS6 | AAGGAGTA | AGGAGTCC |
| 127 | HS7 | AAGGAGTA | CATGCCTA |
| 130 | HS8 | AAGGAGTA | GTAGAGAG |
| 132 | HS9 | AAGGAGTA | CCTCTCTG |
| 134 | HS10 | AAGGAGTA | AGCGTAGC |
| 144 | HS11 | AAGGAGTA | CAGCCTCG |
| 146 | HS12 | AAGGAGTA | TGCCTCTT |
| 151 | HS13 | AAGGAGTA | TCCTCTAC |
| 153 | HS14 | AAGGAGTA | GGTATAAG |
| 154 | HS15 | AAGGAGTA | CAGCTAGA |
| 155 | HS16 | AAGGAGTA | CCATAGCA |
| 157 | HS17 | AAGGAGTA | GGTATAGC |
| 158 | HS18 | AAGGAGTA | GGTTATGC |
| 159 | HS19 | AAGGAGTA | TAGGCAAG |
| 160 | HS20 | AAGGAGTA | TTGTCCAT |
| 162 | HS21 | AAGGAGTA | TCTAGGCA |
| 163 | HS22 | CTAAGCCT | TCGCCTTA |
| 165 | HS23 | CTAAGCCT | CTAGTACG |
| 167 | HS24 | CTAAGCCT | TTCTGCCT |
| 168 | HS25 | CTAAGCCT | GCTCAGGA |
| 170 | HS26 | CTAAGCCT | AGGAGTCC |
| 171 | HS27 | CTAAGCCT | CATGCCTA |
| 172 | HS28 | CTAAGCCT | GTAGAGAG |
| 173 | HS29 | CTAAGCCT | CCTCTCTG |
| 174 | HS30 | CTAAGCCT | AGCGTAGC |
| 176 | HS31 | CTAAGCCT | CAGCCTCG |
| 180 | HS32 | CTAAGCCT | TGCCTCTT |
| 194 | HS33 | CTAAGCCT | TCCTCTAC |
| 197 | HS34 | CTAAGCCT | GGTATAAG |
| 199 | HS35 | CTAAGCCT | CAGCTAGA |
